# Update on food allergy

**DOI:** 10.1111/pai.13443

**Published:** 2021-01-21

**Authors:** Rachel L. Peters, Marta Krawiec, Jennifer J. Koplin, Alexandra F. Santos

**Affiliations:** ^1^ Murdoch Children’s Research Institute Melbourne Melbourne Vic. Australia; ^2^ Department of Paediatrics University of Melbourne Melbourne Vic. Australia; ^3^ Children’s Allergy Service Guy’s and St. Thomas’ NHS Foundation Trust London UK; ^4^ Department of Women and Children’s Health (Paediatric Allergy) Faculty of Life Sciences and Medicine School of Life Course Sciences King’s College London London UK; ^5^ Peter Gorer Department of Immunobiology School of Immunology and Microbial Sciences King’s College London London UK; ^6^ School of Population and Global Health University of Melbourne Melbourne Vic. Australia; ^7^ Asthma UK Centre for Allergic Mechanisms of Asthma London UK

**Keywords:** basophil activation test, biologics, diagnosis, food allergy, IgE, immunotherapy, skin prick test

## Abstract

Food allergy is a major public health issue with growing prevalence in the urbanized world and significant impact on the lives of allergic patients and their families. Research into the risk factors that have contributed to this increase and their underlying immune mechanisms could lead us to definitive ways for treatment and prevention of food allergy. For the time being, introduction of peanut and other allergenic foods in the diet at the time of weaning seems to be an effective way to prevent the development of food allergy. Improved diagnosis and appropriate management and support of food allergic patients are central to patient care with food immunotherapy and biologics making the transition to clinical practice. With the new available treatments, it is becoming increasingly important to include patients' and family preferences to provide a management plan tailored to their needs.

**Key Message**

Food allergy is a major health issue in the urbanized world with increasing prevalence and significant impact on patients' lives. The diagnosis of food allergy is based on clinical history and evidence of allergen‐specific IgE, with oral food challenge being the gold standard and new tests being developed. There is no curative treatment for food allergy, and allergen avoidance is the mainstay of management, with allergen immunotherapy and biologicals being tested currently. The intervention that is widely recommended to prevent food allergy is introduction of peanut and egg at the time of weaning, alongside with breastfeeding.

## 1 IMPACT OF FOOD ALLERGY

Food allergy (FA) can be classified into IgE‐ and non–IgE‐mediated depending on the involvement of IgE in its pathogenesis. In this review, we are focusing on IgE‐mediated food allergy. FA affects about 8% of children in the Western countries and seems to be rising in other parts of the world such as in Vietnam and South Africa, and other parts of Asia and Africa, particularly in urban rather than rural areas.^1^, ^2^, ^3^, ^4^ The prevalence of FA has increased over the recent decades, as has the number of hospitalizations for food‐induced anaphylaxis, following what seems to be the ‘second wave of the allergy epidemic’ after the rise in the prevalence of asthma and respiratory allergy in previous decades.^5^, ^6^, ^7^ Pouessel et al^8^ have shown that foods caused 37% of cases of ICU admissions for anaphylaxis and 79% of recurrent anaphylaxis. Self‐reported FA is even more common with an often underappreciated impact.^1^ Gupta et al^1^ report that about 40% of food allergic children report multiple food allergies, often severe food allergies, and carry an adrenaline auto‐injector. In Western countries, such as the USA and the UK, FA affects disproportionally children from ethnic minorities, such as children of Afro‐Caribbean descent.^1^, ^9^, ^10^ Whether this has to do with genetic predisposition in face of environmental factors related to the modern lifestyle or whether the cultural background, the history of inequality and different access to health care also play a role is unclear.^10^, ^11^ The threefold higher risk of peanut and other food allergies in infants born in Australia to Asian‐born parents compared with the risk of peanut allergy in infants born to Australian‐born parents reinforced the rapidity with which these changes occur and the importance of gene‐environment interactions that need to be further explored.^12^

There is no curative treatment for FA, and the mainstay of management is allergen avoidance. Emergency medication needs to be made available to patients to enable them to treat acute allergic reactions that may result from accidental exposure to the culprit allergens and are unfortunately common.^13^ Allergen avoidance imposes dietary restrictions, with potential nutritional consequences, and can lead to food insecurity.^14^, ^15^, ^16^ Eighty‐six per cent of mothers of children with suspected FA avoid foods on their own initiative.^17^ Goldberg et al^16^ have recently shown that milk‐allergic young adults have reduced bone mineral density and that low calcium intake, asthma and weight constitute independent risk factors. FA can also result in an impairment of quality of life and mental health of children and their families.^17^, ^18^, ^19^, ^20^ For instance, mothers of children with suspected FA have higher state and trait anxiety scores than healthy controls^17^ and about 50% of children and teenagers with FA experience bullying.^18^ FA can also impact negatively on the costs, related to not only the healthcare but also the indirect costs, for instance related to school and work absences, and the financial burden on the families themselves, resulting, for example, from the need to spend more time shopping and to find alternative foods that are often more expensive. All these factors account for additional negative impact on the lives of children with FA and their families that goes beyond the state of hypersensitivity to the culprit allergens, and underscore the importance of an accurate diagnosis and the search for specific treatments for FA.

## 2 EPIDEMIOLOGY

The prevalence of IgE‐mediated FA is highest in infancy and early childhood, driven by a relatively high prevalence of egg and cow's milk allergy that often resolves later in childhood. By contrast, peanut and tree nut allergies, which also typically present in infancy, are less likely to resolve and therefore predominate in later childhood.^21^ Marked differences in the prevalence of FA between countries have been noted for multiple foods, although data from some countries remain sparse.^22^, ^23^, ^24^, ^25^, ^26^ More recent studies have shown that large differences in FA prevalence can exist even within individual countries, with some of this difference driven by a lower prevalence in rural areas compared with urban areas.^4^, ^27^, ^28^ Reasons for these differences are largely speculative, with differences in the prevalence of the risk factors described below potentially playing a role.

The strongest known risk factor for FA is probably eczema, particularly eczema that starts early in life and is more severe.^27^, ^28^ This finding has been noted consistently across studies in both population‐based studies and allergy clinics for many years; however, the mechanism driving this association remains unclear. It has been hypothesized that a damaged skin barrier resulting from eczema may allow the absorption of food allergens through the skin leading to food sensitization and allergy, in the absence of pre‐existing oral tolerance to those foods.^29^ Alternative explanations include the existence of shared genetic or environmental risk factors leading to an increased risk of both eczema and FA.

There has been strong interest in identifying factors that can be modified to prevent FA. Both observational studies and randomized controlled trials have investigated the association between FA and factors including vitamin supplements, fish oil, probiotics and timing of introduction of allergenic foods. These are described further below in the FA prevention section. Other factors that have been associated with risk of FA include factors potentially associated with increased microbial exposure such as pet dogs and older siblings.^30^, ^31^

## 3 MECHANISMS AND PATHOPHYSIOLOGY

The mechanisms underlying IgE‐mediated food allergy is type I hypersensitivity. Understanding the underlying immune mechanism can help us identify targets for treatment and other interventions to prevent and reduce the impact of FA. T cells are central coordinators of the immune response to food allergens, namely the production of antibodies by B cells. Using mass cytometry for immunoprofiling of infants, Neeland et al^32^ described cellular fingerprints associated with peanut allergy and tolerance among IgE‐sensitized infants. Peanut‐allergic infants had increased frequency of CD19^hi^HLA‐DR^hi^–activated B cells and of peanut‐specific memory CD4+ T cells, as well as overproduction of TNF‐alpha, whereas peanut‐sensitized tolerant infants had reduced frequency of CD4+ naïve T cells and an increased frequency of plasmacytoid dendritic cells. Following the description of the new subset of Th2 cells typical of highly allergic patients, the TH2A cells, that decreased following allergen‐specific immunotherapy by Wambre et al,^33^ Chiang et al^34^ found highly differentiated Th2 cells in the peripheral blood of peanut‐allergic patients who were resistant to the countereffect induced by regulatory T cells, whereas healthy controls did not have detectable T‐cell responses to peanut. A stability of T regulatory response was reported by Weissler et al^35^ in both allergic and non‐allergic subjects, with a Th2‐ and Th1‐skewed peanut response detected in sensitized and non‐sensitized individuals, respectively. However, Pellerin et al found that Tr1 cells were functionally impaired in peanut‐allergic patients compared with healthy controls. Ruiter et al^36^ studied the TCR repertoire of CD154+CD4+ memory T cells and found strong convergent selection of peanut‐specific clones that were more numerous among effector T cells of peanut‐allergic patients, with an imbalance between effector and regulatory T cells. The more reactive patients had a more diverse and polarized Th2 effector phenotype with the expression of Th2 cytokines correlating with peanut‐specific IgE levels.

Recently, new studies have shed light on the role of antibodies in allergy and tolerance and on the still puzzling discrepancy between the presence of allergen‐specific IgE and clinical reactivity to foods. For instance, a new subset of T follicular helper cell has been identified in the germinal centre and designated Tfh13 cells.^37^ Tfh13 cells are characterized by a distinct transcription factor profile that includes BCL6 and GATA‐3, and by the production of IL‐4 and Il‐13. Tfh13 result in the production of high‐affinity IgE that is able to induce anaphylaxis to allergens. This high‐affinity IgE is most likely a result of indirect isotype switching from IgG1+ to IgE+ B cells. Contrary to IgG and IgE that depend on germinal centres and Tfh cells, IgA seems to follow an independent mechanism that requires T cells and CD40 ligand but is independent of germinal centres, Tfh and T follicular regulatory cells.^38^ Interestingly, Hoh et al^39^ have shown that the class switch recombination from IgG to IgE and the somatic hypermutation that lead to increased affinity for allergens could develop in the gut of peanut‐allergic individuals, underscoring the importance of gut‐associated lymphoid tissue in FA.

Apart from intrinsic characteristics of IgE, such as affinity for allergens, post‐translational modifications such as glycosylation can have an impact in the ability of IgE to cause effector cell activation and consequently allergic reactions. In a recent study, Shade et al^40^ reported that total IgE from peanut‐allergic subjects had higher sialic acid content compared with non‐atopic subjects and that desialylation of IgE reduced effector cell degranulation and consequent anaphylaxis, raising a new possibility for intervention to treat allergic disease, including FA.

The differences in T‐ and B‐cell and antibody responses between allergic and sensitised tolerant individuals modulate the effector cell response. Hemmings et al^41^ showed that Ara h 2–specific IgE induced greater inhibition of IgE binding and greater mast cell degranulation than Ara h 6, confirming that despite the sequence and structural similarities between Ara h 2 and Ara h 6 and the fact that both are major allergens in peanut, Ara h 2 is the dominant allergen. Effector cell response to allergen can support the identification of phenotypes of food‐allergic patients who may deserve different types of follow‐up and may have indication for specific treatments, such as allergen‐specific immunotherapy or biologics. In a study of egg‐allergic children, changes in the basophil reactivity but not in the T‐cell compartment explained the differences in clinical reactivity to baked egg.^42^ During peanut oral immunotherapy (OIT), Patil et al^43^ assessed basophil responses to Ara h 2 in peanut‐allergic patients at baseline and at different time‐points. Basophil sensitivity, defined by the concentration at which basophils reacted, after 3 months of OIT, could distinguish the patients who responded and had sustained unresponsiveness at the end of the trial from the patients who had transient desensitization and whose basophil response to Ara h 2 rebounded after stopping OIT.

To conclude, understanding the immune mechanisms underlying FA and oral tolerance is key to improve diagnostics and the care for patients and their families and identify targets for a definitive treatment of FA. Table 1 summarizes recent new discoveries about immune mechanisms of FA.

**TABLE 1:** Highlights of new discoveries about immune mechanisms of food allergy

T cells and T follicular helper cells	•Food allergy involves Th2‐skewed response more than a dysregulated regulatory T‐cell population.^34^, ^35^ •The new subset of T follicular helper cells designated Tfh13 induces the sequential class switching from IgG1 to IgE, leading to the production of high‐affinity IgE that can cause anaphylaxis.^37^
B cells and antibodies	•IgE class switching can happen in the gut‐associated lymphoid tissue.^39^ •IgA induces tolerance through immune exclusion rather than active suppression and is generated via a separate mechanism that is independent of Tfh and germinal centres.^38^
Basophils and mast cells	•IgE glycosylation enhances effector cell degranulation.^40^ •Basophil response to allergen can distinguish responders from non‐responders as early as 3 months into oral immunotherapy.^43^

## 4 DIAGNOSIS

An accurate diagnosis of FA is essential. Correctly identifying FA is crucial for providing education and management strategies to mitigate the risks of a potentially life‐threatening allergic reaction. In contrast, correctly identifying food tolerance will promote dietary liberation, which is especially important in the light of the paradigm shift encouraging early introduction of allergenic foods to prevent FA.^44^ Double‐blind placebo‐controlled food challenges remain the gold standard for FA diagnosis. Updated guidance on performing oral food challenges has recently been published, with additional focus on safety, psychosocial considerations, and baked egg and milk challenges, to name a few.^45^ However, due to the inherent risks and intensive resource requirements, their feasibility is limited in some clinical and research settings. The utility of traditional tests of sensitization (SPT and sIgE), as well as development of new molecular techniques that are able to diagnose food allergy without the need for oral food challenges, remains an active area of research. This section highlights recent advances in this area.

Skin prick tests (SPT) and serum‐specific IgE (sIgE) are routinely used in clinical practice and are relatively safe and inexpensive to perform. However, the conventional positive results (SPT ≥ 3 mm or sIgE ≥ 0.35 kU/L) have poor specificity to clinical FA, with approximately half of sensitized individuals able to tolerate the food without reaction. As increasing magnitude of these tests correlates with a higher risk of reaction, many studies have defined thresholds for these tests with 95% positive predictive value (PPV) to FA (reviewed in^46^, ^47^, ^48^, ^49^, ^50^, ^51^). Although SPT and sIgE thresholds with 95% PPV to FA are routinely used to minimize the need for diagnostic food challenges, a proportion of children remain in the immunologic grey area; that is, they are food‐sensitized but below the 95% PPV threshold. New approaches that can accurately diagnose FA while reducing the need for food challenges are urgently needed.

Allergen component‐resolved diagnostics (CRD) are proposed as a more accurate method of diagnosis, because instead of using crude allergen extracts, which consist of both allergenic and non‐allergenic components, CRD measures sIgE to individual allergen proteins. A systematic review comparing SPT and sIgE to whole peanut and its components concluded that sIgE to Ara h 2 had greater diagnostic accuracy compared with the other tests.^49^ Furthermore, a meta‐analysis of 19 studies found that while sIgE to Ara h 1, Ara h 2 and Ara h 3 had high specificity to peanut allergy, sensitivity was highest in Ara h 2. The pooled sensitivity and specificity of Ara h 2 ≥ 0.35 kU/L to peanut allergy were 83% (95% CI 76%‐89%) and 84% (95% CI 77%‐88%).^52^ Likewise, further studies support that CRD offer greater accuracy compared with sIgE to whole allergens for hazelnut^53^ and it is plausible that this increased accuracy applies to other foods. The major allergen components for most common food allergens have been isolated, and research continues to identify the optimal cut‐off points.^54^

Approaches to the diagnosis of FA using cellular tests also appear to offer greater sensitivity and specificity than traditional tests. The basophil activation test (BAT) measures the expression of activation markers on the surface of basophils, stimulated with food allergens and controls, by flow cytometry.^55^ In a study of 104 children, BAT demonstrated superior ability to discriminate between peanut‐allergic and peanut‐sensitized tolerant children compared with SPT, sIgE and sIgE to Ara h 2. The optimal diagnostic parameter and threshold demonstrated an impressive sensitivity and specificity of 98% (95% CI 87‐100) and 96% (95% CI 86‐100), respectively. BAT performed similarly well when validated in an independent sample (83% sensitivity and 100% specificity).^56^ For other allergens, BAT performed well but not necessarily superior to other measures. In a prospective study of 83 children with suspected tree nut allergy, SPT demonstrated greater sensitivity to BAT, while BAT demonstrated greater specificity compared with SPT; AUC was similar for both measures with the exception of hazelnut where BAT had greater AUC than SPT.^57^ While the performance of BAT appears promising, its clinical utility may be limited because it requires live cells and flow cytometry equipment. BAT may therefore be more feasible in settings where it can be used in combination with conventional diagnostic tests. For example, performing peanut BAT as a second step following equivocal SPT or sIgE to Ara h 2 reduced the need for OFC by 97% compared with the combination of SPT and sIgE to whole peanut.^56^

The mast cell activation test (MAT) offers another promising approach and has the advantage over BAT that it uses stored plasma rather than fresh whole blood. In the same sample as described previously for peanut BAT,^56^ MAT performed equally well to BAT in terms of specificity; however, the sensitivity of MAT was lower than BAT.^58^ Importantly, MAT provided definitive results in all cases where basophils were non‐responsive.^58^ In a smaller study, MAT performed better than BAT based on AUC for the diagnosis of peanut allergy; however, confidence intervals overlapped.^59^ The utility of these tests has been assessed for some other common allergens and performs similarly well but further research is needed.^60^ Additionally, these cellular tests may offer additional clinical utility as the results are correlated with reaction severity,^59^, ^60^ whereas SPT and sIgE are not always predictive of reaction severity.^61^, ^62^ However, further work is required to inform standardization of laboratory procedures, optimal test parameters and thresholds, and cost‐effectiveness in different settings before these novel approaches are ready for routine clinical practice.^55^

**FIGURE 1:**
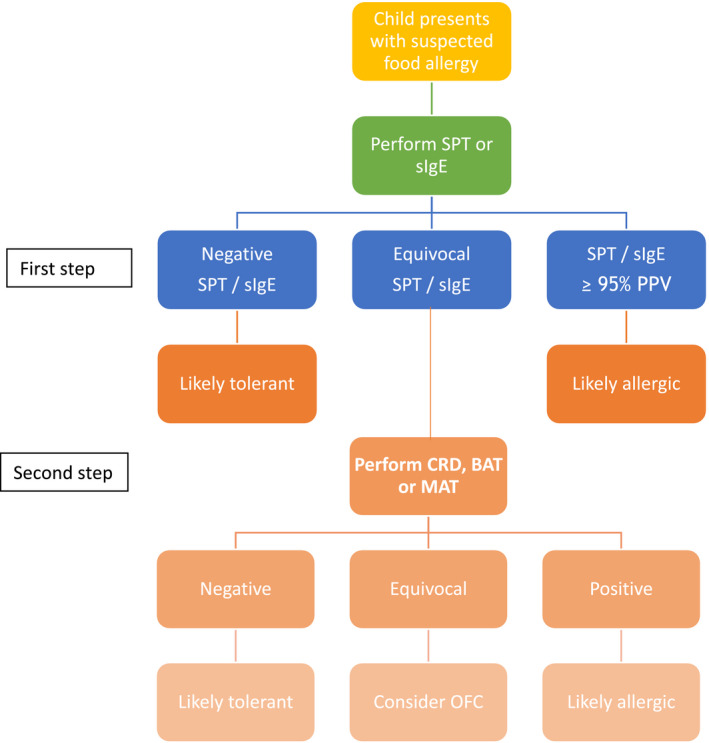
Proposed use of component‐resolved diagnostics (CRD), basophil and mast cell activation tests (BAT and MAT) in combination with conventional tests, skin prick test (SPT) and specific IgE (sIgE), to reduce the need for oral food challenges (OFC)

Despite continued advances and development of novel molecular techniques, identifying a definitive diagnostic test to negate the need for oral food challenges remains elusive. The optimal threshold requires a trade‐off between false negatives and false positives, and this varies in the published literature due to heterogeneity in study sample, design, methods, regional characteristics, allergen extracts and laboratory procedures. Figure 1 represents a suggested approach to the sequential use of diagnostic tests to improve the diagnosis of food allergy without the need for OFC, as proposed by several studies.^63^ This approach involves first‐line tests of traditional SPT and/or sIgE using established 95% PPVs. If results are equivocal, a second‐line test of CRD, BAT or MAT may be ordered and this approach has been shown to substantially reduce the need for OFC.^63^ However, OFC remain the gold standard and may be required to confirm the diagnosis if all tests are equivocal. Identification, validation and cost‐effectiveness of the optimal diagnostic approach for FA continue to be an active area of research.

## 5 TREATMENT

### 5.1 Allergen avoidance

In the absence of effective treatment, allergen avoidance and providing appropriate emergency medication used to be the only approach to management of FA.^64^ Avoidance of food allergen is onerous for patients and families and often fails with ten per cent of patients on average experiencing an allergic reaction per year.^65^, ^66^, ^67^ Additionally, allergen avoidance inflicts multiple pressures on allergic individuals and their families, food manufacturers, and restaurants and public spaces such as schools and aircrafts.^68^, ^69^ Precautionary allergen labelling is in general voluntary and used inconsistently across industry which can be misleading for patients and caregivers.^65^

Providing adrenaline auto‐injectors (AAI) to patients at risk of anaphylaxis encounters challenges related to their availability, which is mostly limited to high‐income countries, varied national regulations in prescribing and high cost.^70^ When prescribed, AAI are only carried at all times by half of the patients^71^ and mistakes in use are frequent among both patients^72^ and medical staff.^73^

Meeting the needs of both food‐allergic children undergoing immunotherapy and those continuing strict avoidance in the same environment, for example school or household with two allergic siblings managed differently, is an arising challenge.

### 5.2 Food immunotherapy

**FIGURE 2:**
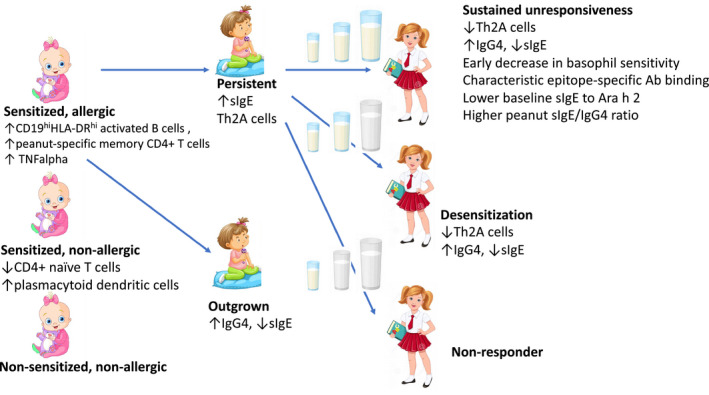
Clinical phenotypes of food‐sensitized and food‐allergic children and possible outcomes of food immunotherapy. Although the largest evidence comes from peanut studies, the concepts highlighted here are applicable to other food allergies

Just over twenty years since the first RCT demonstrated its efficacy,^74^ food immunotherapy (FIT) has become the first established treatment modality for FA, which is now recognised by national and international guidelines.^75^, ^76^, ^77^ The efficacy of oral FIT has been documented in RCT in children with milk, egg and peanut allergy,^78^ with lower desensitization rates being achieved in wheat allergy.^79^ In the largest oral FIT study so far, the PALISADE study, which investigated efficacy of 300‐mg dose of peanut protein in inducing tolerance to peanut in almost 500 children ≥ 4 years, 67.2% of participants achieved the primary end‐point of passing 600‐mg dose at the exit DBPCFC.^80^ It has also been confirmed recently in a placebo‐controlled study that peanut oral IT (POIT) significantly reduces the risk of reaction after accidental exposure to peanut (placebo group, 24 reactions in 14 patients; active group, eight reactions in five patients; *P* < .001).^81^ Nevertheless, the recent safety meta‐analysis, which looked into 12 POIT studies, estimated that the risk of anaphylaxis while on POIT is over three times higher compared with peanut avoidance (RR, 3.12, 95% CI 1.76‐5.55) and the risk of adrenaline use is over twice as high (RR, 2.21; 95% CI 1.27‐3.83).^82^ Therefore, the current focus of FIT research is orientated towards answering crucial questions about increasing safety of FIT by choosing well‐tolerated and effective formulation,^83^ route and dose, adding adjuvants at the initial stage of the treatment and identifying patients most likely to benefit from FIT. The two most studied alternative routes to oral FIT are sublingual (SLIT) and epicutaneous IT (EPIT). Their safety profile is favourable with few systemic allergic reactions reported; it comes, however, at the cost of lower efficacy.^84^, ^85^, ^86^, ^87^ The modest level of desensitization predisposes SLIT and EPIT for use in individuals not tolerating OIT.^87^ It may also be the case that longer treatment duration is necessary to achieve results comparable with OIT.^84^ The other main need is understanding long‐term outcomes of the treatment.^88^, ^89^ Table 2 summarizes recent developments in FIT, and Figure 2 illustrates phenotypes of food allergy and possible outcomes of FIT.

**TABLE 2:** Recent developments in food immunotherapy (FIT)

Route	In the large phase 3 study on epicutaneous IT to peanut, 35.3% of participants achieved predefined response rate compared with 13.6% of children in placebo group; despite the difference being statistically significant, the 95% CI exceeded pre‐specified lower cut‐off, which means the study did not meet its primary end‐point.^102^
Dose	Daily dose equivalent of one peanut and ten peanuts exert similar clinical and immunologic effects in peanut IT in young children.^103^ No use of adrenaline related to treatment was reported in the recent peanut OIT study in which maintenance peanut protein dose was established at a low dose (between 125 mg and 250 mg).^81^ In the group of Japanese children with history of anaphylaxis to wheat, 31% of subjects developed mild anaphylaxis despite low‐dose protocol (53 mg of wheat protein).^104^
Age	FIT tends to be associated with reassuring safety profile and higher rates of sustained unresponsiveness if started early.^103^ In the Italian cohort of 73 infants with IgE‐mediated milk allergy who underwent milk OIT, 97% reached the target 150‐mL dose of milk. No patient required use of AAI at home.^105^
Formulation	The BOPI study looked into effectiveness and safety of boiled peanut IT. 28% of participants presented with 1.9 episodes of anaphylaxis during treatment, which is comparable to average rate of severe adverse events reported in other studies. Small proof‐of‐concept study confirmed that baked egg IT led to desensitization to lightly cooked egg with no moderate or severe adverse events noted.^Eg^ Egg IT is more effective in inducing sustained unresponsiveness than baked egg consumption.^107^
Adjuvants	Multiple adjuvant agents have been tested in the context of improving benefit‐risk ratio in FIT, from probiotics and Chinese herb medicine through montelukast and antihistamines to biologic treatments.^108^ Omalizumab allows quicker up‐dosing with fewer adverse events without affecting immunologic desensitization processes.^108^ Omalizumab may potentially mask early symptoms of gastrointestinal disease related to FIT.^110^ Adverse events may start occurring after discontinuation of anti‐IgE during the maintenance phase.^111^, ^112^
Sustained unresponsiveness	The baseline epitope‐specific antibody binding models can achieve even 87% accuracy in predicting SU in milk OIT.^113^ In peanut oral IT, early decrease in basophil sensitivity to Ara h 2 correlates with SU.^43^ Higher baseline peanut‐specific IgG4‐to‐IgE ratio and lower Ara h 2 IgE and basophil activation responses were associated with sustained unresponsiveness in the POISED study.

Despite the efficacy in inducing desensitization to the culprit food, the outcome of FIT differs from natural outgrowing of FA. While the benefits of a margin of protection in case of accidental exposure and introducing certain amount of the food in regular diet are possible during the treatment, the long‐term effect remains unpredictable with up to 70 per cent successfully desensitized individuals losing tolerance after a short period of avoidance.^43^ Why the post‐IT tolerance is lost despite apparent similarities in immunologic response with FA resolution (e.g. decrease in specific IgE concentration and raise in specific IgG4) remains unclear.^90^

As sustained unresponsiveness is not achieved by at least half of the patients, the question about the necessary frequency of consumption of the food after completion of FIT remains. Reassuringly, consumption of an egg twice a week has proven sufficient to sustain tolerance in the Spanish SEICAP study.^91^ In the large long‐term follow‐up Finnish cohort of children who completed milk OIT, only a quarter of the children returned to milk avoidance diet during the median 6.5‐year‐long observation period.^92^ Regarding ongoing peanut consumption, 64% of previous peanut IT participants continued to ingest peanut daily and another 25% less frequently. Unfortunately, allergic reactions including airway involvement were still noted even in this late stage of desensitization.^93^ With the first commercial product for peanut OIT approved by FDA in January 2020, FIT is likely to become more widely available and uniform in the coming years.

### 5.3 Biologicals

In FA, biologic treatments have been mostly investigated in the context of facilitating FIT. In addition to the above‐mentioned FIT/anti‐IgE studies, which have already been completed, there are ongoing projects looking at use of dupilumab in combination with peanut OIT (Clinicaltrials.gov NCT03793608, Clinicaltrials.gov NCT03682770), combination of dupilumab and omalizumab in multi‐food OIT (Clinicaltrials.gov NCT03679676), and anti‐IL‐33 in peanut OIT (Clinicaltrials.gov NCT02920021).^94^

Due to its pathomechanism, eosinophilic pathway inhibition has been extensively studied in the treatment of EoE.^95^ The use of anti‐IL‐5, anti‐IL‐13 and anti‐IL‐4 has been associated with significant reduction in histologic features of EoE in three RCTs.^96^, ^97^ However, there have been no clear clinical improvement noted. Therefore, the treatments are currently not routinely recommended in EoE management.^98^

Recently, inhibition of alarmins (IL‐25, IL‐33 and TSLP) in a mouse model was effective in preventing FA,^99^ which may suggest future promising direction of biologic use in FA.

## 6 PREVENTION

Despite significant progress in identifying risk factors for FA, there is still little that can be recommended to prevent FA. Few of the known risk factors described above are easily modifiable. Furthermore, of the potentially modifiable factors tested in clinical trials to date, most have not been effective in preventing FA. A recent systematic review by the European Academy of Allergy and Clinical Immunology FA and Anaphylaxis Guidelines Group^100^ identified 41 randomized controlled trials of potential FA prevention strategies in infancy and childhood. The vast majority of these trials showed little to no effect on preventing FA, including trials of dietary avoidance of food allergens, vitamin supplements (maternal and infant), fish oil, probiotics, prebiotics, symbiotics and hydrolysed formulas. However, the authors also concluded that the evidence around most of these interventions remains very uncertain. Many of the trials were at risk of bias due to lack of robust diagnostic criteria, high loss to follow‐up, potential confounding, and lack of blinding, and were underpowered for the outcome of interest.

Although some of the risk of FA is likely to be already established at birth, to date there are no known effective preventative strategies that can be applied during pregnancy. The only intervention that is currently widely recommended to reduce the risk of FA is timely introduction of peanut into the infant's diet. This recommendation is primarily based on the results of a large, high‐quality randomized controlled trial in high‐risk infants conducted in the United Kingdom^9^—a country with a relatively high prevalence of FA. The relevance of these findings to countries with a low peanut allergy prevalence is less clear.^101^ There is also evidence from meta‐analyses of multiple trials that early introduction of egg into the infant diet reduces the risk of egg allergy, although the extent of the reduction in risk appears lower than for peanut.^44^

## 7 CONCLUSION

Food allergy is a major public health issue with growing prevalence in the urbanized world and significant impact on the lives of allergic patients and their families. Research into the risk factors that have contributed to this increase and their underlying mechanisms could pave the way to definitive ways for treatment and prevention of FA. For the time being, introduction of peanut and other allergenic foods in the diet at the time of weaning seems to be an effective way to prevent the development of FA. Improved diagnosis and appropriate management and support of food‐allergic patients is central to patient care with food immunotherapy and biologicals making the transition to clinical practice. With the new available treatments, it is becoming increasingly important to include patient's and family preferences to provide a management plan tailored to their needs.

## CONFLICTS OF INTEREST

Alexandra F. Santos reports grants and personal fees from Medical Research Council (MR/M008517/1; MR/T032081/1); grants from Food Allergy Research and Education (FARE), Asthma UK and the NIHR through the Biomedical Research Centre (BRC) award to Guy's and St Thomas’ NHS Foundation Trust, during the conduct of the study; grants from Immune Tolerance Network/National Institute of Allergy and Infectious Diseases (NIAID, NIH); grants from Asthma UK; personal fees from Thermo Scientific, Nutricia, Infomed, Novartis, Allergy Therapeutics, and Buhlmann; and research support from Buhlmann and Thermo Scientific through a collaboration agreement with King's College London. RLP and JK receive research support from the National Health and Medical Research Council of Australia. The other authors declare no conflicts of interest.

## AUTHOR CONTRIBUTION

**Rachel Louise Peters:** Writing‐original draft (equal); Writing‐review & editing (equal). **Marta Krawiec:** Writing‐original draft (equal); Writing‐review & editing (equal). **Jennifer Julia Koplin:** Writing‐original draft (equal); Writing‐review & editing (equal). **Alexandra Santos:** Conceptualization (equal); Writing‐original draft (equal); Writing‐review & editing (equal).

### Peer Review

The peer review history for this article is available at https://publons.com/publon/10.1111/pai.13443.
